# Assessing the impact of an English national initiative for early cancer diagnosis in primary care

**DOI:** 10.1038/bjc.2015.43

**Published:** 2015-03-03

**Authors:** G Rubin, C Gildea, S Wild, J Shelton, I Ablett-Spence

**Affiliations:** 1 School of Medicine, Pharmacy and Health, Wolfson Research Institute, Durham University, Queen's Campus, Stockton-on-Tees TS17 6BH, UK; 2 PHE Knowledge and Intelligence Team (East Midlands), 5 Old Fulwood Road, Sheffield S10 3TG, UK; 3 Care Quality Commission, 103-105 Bunhill Row, London EC1Y 8TG, UK

**Keywords:** quality improvement, referral, general practice, leadership

## Abstract

**Background:**

The *Cancer Networks Supporting Primary Care* programme was a National Health Service (NHS) initiative in England between 2011 and 2013 that aimed to better understand and improve referral practices for suspected cancer.

**Methods:**

A mixed methods evaluation using semi-structured interviews with purposefully sampled key stakeholders and an analysis of Cancer Waiting Times and Hospital Episode Statistics data for all 8179 practices in England were undertaken. We compared periods before (2009/10) and at the end (2012/13) of the initiative for practices taking up any one of four specified quality improvement initiatives expected to change referral practice in the short to medium term and those that did not.

**Results:**

Overall, 38% of general practices were involved in at least one of four quality improvement activities (clinical audit, significant event analysis, use of risk assessment tools and development of practice plans). Against an overall 29% increase in urgent cancer referrals between 2009/10 and 2012/13, these practices had a significantly higher increase in referral rate, with reduced between-practice variation. There were no significant differences between the two groups in conversion, detection or emergency presentation rates. Key features of successful implementation at practice and network level reported by participants included leadership, organisational culture and physician involvement. Concurrent health service reforms created organisational uncertainty and limited the programme's effectiveness.

**Conclusions:**

Specific primary care initiatives promoted by cancer networks had an additional and positive impact on urgent referrals for suspected cancer. Successful engagement with the programmes depended on effective and well-supported leadership by cancer networks and their general practitioner (GP) leads.

The English National Awareness and Early Diagnosis Initiative (NAEDI; Richards, 2009) was launched in 2008, in response to the Cancer Reform Strategy (Department of Health, 2007), in order to understand and address the reasons for late diagnosis of cancer in England. One of its four work streams aimed to overcome clinical and system barriers to prompt onward referral within and between primary and secondary care, with a focus on general practitioner (GP) awareness of symptoms and interaction with patients, the interface between primary and secondary care, including any disincentives to referral, and the commissioning and ‘gatekeeper' function of GPs (Cancer Research UK, 2010). The National Awareness and Early Diagnosis Initiative is a public sector/third sector partnership, led by National Health Service (NHS) England, Public Health England, Cancer Research UK and the Department of Health. Until April 2013, The National Cancer Action Team (NCAT) played a key role, on behalf of NHS England, in its implementation.

Early NAEDI initiatives in primary care included a National Audit of Cancer Diagnosis (Rubin *et al*, 2011) and the development of a significant event analysis template applicable to cancer diagnosis (Mitchell *et al*, 2012). The role of a GP lead within a cancer network was developed and systematically implemented throughout England, initially to support the introduction of General Practice Profiles for Cancer in 2010 (National Cancer Intelligence Network, 2014). Following the publication *of Improving Outcomes: a Strategy for Cancer* (Department of Health, 2011), a programme of centrally coordinated, locally delivered improvement initiatives commenced, titled *Cancer Networks Supporting Primary Care*. As this programme progressed, its focus narrowed down to those initiatives that key stakeholders felt were having the greatest impact, notably, but not exclusively, review of practice profiles for cancer and development of practice action plans, use of risk assessment tools, clinical audit and significant event analysis (Box 1). GP leads were instrumental in encouraging practices to take up one or more of these activities.

During the same period, the English NHS was the subject of major organisational reform, much of which became operational in April 2013 (Department of Health, 2010). As a result, cancer networks were subsumed into a smaller number of strategic clinical networks, with a remit for quality improvement that embraced multiple clinical areas in addition to cancer.

The evaluation of large-scale quality improvement initiatives, particularly those in which the implementation of a national policy direction is locally determined, presents organisational and methodological challenges. Circumstances, capacity and timescale may preclude an optimal design, whereas choice of methods and outcome measures can influence the utility of findings (Salisbury *et al*, 2010). There are few reported evaluations of large-scale transformational change in the peer-reviewed literature. A mixed method approach has been adopted for some comparable policy-driven initiatives, notably the evaluation of the Department of Health Integrated Care Pilots (Ling *et al*, 2010), whereas realist evaluation has been increasingly used to better understand the complex relationships between context, mechanisms and outcomes – for example, in the London Modernisation Initiative (Greenhalgh *et al*, 2009). One of the most significant quality improvement initiatives in the UK, ‘Pay for Performance', has been the subject of a programme of research utilising a wide range of methodologies in multiple individual studies (Gillam *et al*, 2012). For this evaluation, we aimed to understand the context in which the programme was implemented, the mechanisms used to effect its introduction and the extent to which its objectives were achieved.

## Materials and methods

A logic model for the programme, constructed with the help of key stakeholders, informed our evaluation (Supplementary Online Material and Supplementary Figure 1). We adopted a mixed methods approach to evaluation. We undertook serial one-to-one semi-structured interviews with a purposive sample of key individuals, identified by NCAT and Cancer Network Directors, based on their knowledge of a person's involvement in the development and delivery of the programme. An interview guide was developed from the Logic Model and used on each occasion. Interviews were recorded and were professionally transcribed. Transcripts were read and re-read independently by two members of the research team and a coding frame constructed, which was then applied to the data to identify emerging themes. We also undertook documentary analysis of meeting notes, activity logs, practice plans and other materials used by the networks. Analysis drew on principles of realist evaluation (Pawson and Tilley, 1997) to develop a contextualised account of the way in which change emerged, initially considering the individual components of the logic model (Ablett-Spence *et al*, 2012, 2013). In this paper, we report the cross-cutting themes that emerged from that analysis. We also compared our findings against the contextual barriers and facilitators to quality improvement identified in a recent systematic review (Kaplan *et al*, 2010).

Because impact on referral practice was identified as a key outcome for the programme, we compared referral activity in the 12 months to March 2010, that is, before the start of the programme, with the same period to March 2013. We hypothesised *a priori*, in consultation with NCAT, that four specific activities (clinical audit, significant event analysis, use of risk assessment tools and practice plans for cancer) would change clinical and referral practice in the short to medium term, and compared those practices that chose to engage at any point in one or more of these against those that did not engage in any of the four.

From Cancer Waiting Times (CWT) data, we obtained the number of urgent GP referrals for all suspected cancers from April 2009 to March 2013, based on ‘date first seen', and the number of cancers receiving a first treatment during the same period, based on ‘treatment start date'. These data were used to calculate referral rate, conversion rate (percentage of urgent GP referrals resulting in a cancer diagnosis) and detection rate (percentage of CWT recorded cancers resulting from an urgent GP referral).

A validated algorithm (National Cancer Intelligence Network, 2013) was applied to Hospital Episode Statistics (HES) patient care data to identify new cancer cases and their mode of presentation and then to calculate the percentage of HES-identified cancers first presenting as an emergency. Emergency presentation was defined as emergency in-patient admission from an A&E department or an outpatient clinic or a GP or Bed Bureau referral, or referral to outpatients following A&E attendance or emergency admission (National Cancer Intelligence Network, 2013).

We examined data for all practices present in the 2009/10 Quality and Outcomes Framework (QOF) data set. On the basis of 2009/10 and 2011/12 QOF list sizes and Attribution Data Set populations from 2009 and 2012, practices with significant changes in practice list size (>10% change in practice population within the considered period) and very small practices (<1000 population) were excluded from the analyses.

### Statistical methods

Referral rates were directly age-standardised (Fay and Feuer, 1997). For the percentage change in referral rate, 95% confidence intervals were derived by transformation of confidence intervals for the rate ratio. For individual GP practices, indirectly age- and sex-standardised referral ratios (SRR) were calculated, based on the number of referrals expected given the practice population structure and the age- and sex-specific referral rates for England, with an SRR of 100 representing the expected referral rate.

The statistical significance of changes, from the before to after period, was determined by means of hypothesis tests, with a considered significance level of 95%. For age-standardised referral rates, the *z*-tests considered the null hypothesis that the ratio of the before and after period referral rates was equal. For GP practice level rates, Brown and Forsythe (1974) tests considered the null hypothesis that there was the same variance in the rates in the before period as in the after period.

## Results

### Referral activity

We obtained data on cumulative activity in relation to the programme from all 28 cancer networks in England and for 8179 practices. For eight networks (2374 practices), we received activity data up to March 2012 only. Of practices in the 2009/10 QOF data, 1819 were removed from the analysis; 537 of these practices had taken up a specified activity. A further 27 practices with a record of at least one specified activity were excluded because they were not in the 2009/10 QOF data.

By March 2013, 2495 (38%) of the practices included in the analysis had taken up one or more of the specified activities. Practices taking up any of these activities had larger list sizes than those that did not and were less likely to be serving deprived areas (Table 1).

Against an overall 29% increase in 2-week referral rates between 2010 and 2013, there was a significantly greater increase for practices engaging in any of the four specified activities, but no significant differences in conversion, detection or emergency presentation rates (Table 2). There were no consistent and significantly greater changes for those practices that engaged in more than one activity. In the period under study, the overall conversion, detection and emergency presentation rates all changed significantly. The conversion rate fell by 1.3 percentage points to 10.2% and the detection rate rose 3.9 percentage points to 47.8%. Emergency presentations fell from 23.4% to 21.1%. There was a significant reduction in the variability of referral ratios and conversion, detection and emergency presentation rates, with a notably larger reduction in the interquartile range of referral ratios for ‘activity' practices compared with those not taking up any specified activity (Table 3). In subanalyses, those practices taking up risk assessment tools showed a significantly greater increase in referral rate for suspected colorectal cancer but not for suspected lung cancer. Conversion and detection rates were not significantly different (Supplementary Online Material; and Supplementary Tables 1 and 2).

### Qualitative findings

We conducted interviews with 92 participants, 38 of whom were interviewed twice and 7 were interviewed three times. They included GPs, GP cancer leads, public health specialists and cancer network staff. Thirty-two participants declined a second interview because they felt they had nothing new to add. Fifteen participants were interviewed for a first time because they had taken the place of an earlier interviewee who had left that post.

### Organisational context and culture

A key contextual factor was the structure of cancer networks and, latterly, the ease or otherwise with which they managed the transition to being part of geographically larger and more clinically diverse strategic clinical networks. A major challenge was to keep engaged those individuals whose role or job security was threatened by this reorganisation:

‘It's been really difficult to implement things, people aren't motivated when their jobs aren't secure and it's hard to speak to key people, as staff are moving into different posts or leaving all the time. There is just no continuity.' – Network Director

At the general practice level, interviewees reported a diversity of practice cultures that demanded flexibility in approaches to engagement. Networks that tried a ‘one size fits all' approach reported less success in implementing quality improvement initiatives.

### Leadership

The programme had consistently strong leadership from the NCAT, with excellent communication and a supportive approach allied to regular monitoring of progress. In cancer networks, effective leadership was associated with organisational stability and consistent messaging. When this was the case, participants reported clinicians feeling better supported to sustain their improvement activities, better engagement with secondary-care clinicians and improvement initiatives focussed on the primary/secondary care interface.

Networks with strong and proactive GP leads reported better engagement with practices, and had greater success in implementing and sustaining initiatives, attributing this to their leads being able to articulate professional and personal relevance to their peers.

### Support

Network GP leads reported that administrative and project management support was key in enabling them to function efficiently. In the early stages of the programme, this was patchy and those who lacked it reported being less effective in engaging with their GP colleagues. Moreover, the type of support varied greatly, from a small amount of secretarial support to a project manager.

Some networks developed a Primary Care Facilitator role to complement their GP lead. Although facilitators and GP leads often reported that the role needed to be validated by the GP lead to be seen as credible by practices, they were highly valued by GP and network programme leads for the additional capacity and skills they brought:

‘So their role was about engaging the whole practice team, about going in, about following up and maybe exploring in a bit more detail some of the issues that the GP (lead) had discussed. Because say you only had an hour, it doesn't always get through, people need to come back and explore it, and particularly things like the risk assessment tool might need two or three goes for people to understand.' – Network Programme Lead

### Communication

Communicating consistent messages in relation to the programme, across a cancer network made up of multiple organisations, each with their own systems, processes and cultures, made implementation difficult. Newsletters were common, but other methods including podcasts and videos were also tried. Established methods of communication were usually more effective than creating something new. There were also concerns about information overload in general practice:

‘I mentioned it (information pack) at a practice visit and they just looked blank. It turns out they had never seen it and it was in the practice library or something, but when they found it and looked at it they thought it was really useful.' – GP lead

### Education and training

All networks invested significant time and effort into providing training in cancer awareness and diagnosis to GPs and other primary care professionals. In general, attendance was better when such training was incorporated in formal protected learning sessions than when provided on an *ad hoc* basis. Although a range of teaching methods were utilised, a range of approaches kept training interesting and different:

‘So for example, I've created a short video about how to use digital cameras (to monitor pigmented lesions); how to upload it on to clinical systems etc. So for us, video is becoming a more integral part of how we offer resources. So as well as paper and online we also offer video and nowadays, the interactive PDF.' – GP lead

Although education in itself was perceived as being important, the discussion that it generated was considered even more valuable by GP and network leads, often signalling a commitment to actively engage with the programme. Practices that were the hardest to engage with the programme were often those whose practitioners did not attend education and training sessions.

## Discussion

A national initiative to improve early detection of cancer was associated with considerable overall improvement in referral practice. Four specific primary care initiatives promoted by cancer networks had an additional and positive impact and reduced between-practice variation. Success in involving practices with the programme was associated with strong clinical and organisational leadership, good clinical engagement with education and training, and a supportive organisational culture. Concurrent health service reforms had a destabilising effect, notably when small leadership teams lost key personnel.

### Strengths and limitations

It is remarkable that we were able to obtain activity data for all practices in England, while the CWT database and HES data are well understood and reliable sources. We used a wide range of informants and serial interviews in order to develop our understanding of the implementation of the programme as it progressed. We were strongly supported by the NCAT in obtaining access to key informants. GP leads in cancer networks were instrumental in our obtaining highly complete data on practice participation in the specific initiatives associated with the programme.

We used realist evaluation as our methodological approach to analysis for the qualitative data. This theory-driven approach helps to illuminate if and why certain elements of an initiative resulted in particular outcomes and aids the understanding of ‘what worked for whom and in what circumstances'. It is being increasingly recognised as a valuable means of understanding how particular preconditions make intended outcomes more or less likely (Pawson and Tilley, 1997).

This evaluation was heavily dependent on individuals in networks, both for data collection and for participation in interviews. Because of the NHS reforms, a number of them lost or changed their jobs during the period of the study. This contributed to us only receiving interim practice activity data from eight cancer networks.

The views of interview participants may not be representative of the range of views held among those involved in the programme's implementation. Although we were able to sample from lists of key stakeholders provided to us by NCAT and Cancer Network directors, both had an interest in the programme succeeding and we cannot exclude attribution or social desirability bias. However, we did encounter negative as well as positive perspectives in the course of our interviews, and a number of our participants were aware that their future lay elsewhere. Indeed, our decision to undertake serial interviews was affected by some loss of continuity of key informants and related loss of corporate memory.

Our analysis of routinely collected activity data has some limitations. The detection rate is based only on cases recorded in the CWT data, rather than on a complete record of cancer registrations, and the emergency presentation rate is based only on cases identified from the HES data. The periods chosen for study are not exclusively before and at the end of all initiatives; a number of clinical audits were started and some completed before March 2010, but this is the earliest year for which reliable and complete CWT data are available. For reasons of data availability, HES data were considered for the 12-month periods between December 2009 and December 2012.

We did not obtain data on the timing of uptake of initiatives by individual practices, nor were we able to measure the quality or completeness of their participation. As such, the extent to which subsequent referral practice could be influenced by participation (the ‘dose effect') will have varied between practices.

### Comparison with other research

Our findings are broadly consistent with those of a recent systematic review of factors influencing quality improvement success in health care (Table 4; Kaplan *et al*, 2010). In particular, we found that leadership from senior management and clinical engagement were important facilitators, and that organisational culture, under challenge from concurrent health service reforms, could be an important barrier. However, we also identified poor communication as a key barrier, whereas training and education, particularly for its ability to stimulate the practice as a team to action, was a powerful facilitator.

In the evaluation of a comparably large-scale initiative of Integrated Care Pilots in England, failure to actively gain participation of key staff groups, such as GPs, made progress difficult (Ling *et al*, 2012). In the United Kingdom, GPs are comparatively autonomous, with no strong overarching management structure. Implementing change presents unique challenges; who manages this change and how it is introduced are key to it being accepted by professionals who may feel their professional autonomy is under threat. The key influencing role played by GP leads in this programme can be interpreted as ‘soft coercion' (Sheaff *et al*, 2003), a form of leadership seen in ‘soft bureaucracies', organisations characterised by a largely autonomous professional group, in this case GPs, which is part of a structure that has a rigid framework and provides governance (Courpasson, 2000).

There is limited literature reporting interventions in primary care to modify referral for suspected cancer. A recent systematic review of interventions to reduce primary care delay in cancer referral identified four studies with relevant referral outcomes (Mansell *et al*, 2011). Two had a demonstrable effect: the first found that an educational intervention for suspected prostate cancer reduced referrals, whereas an RCT of dermoscopy and a three-point checklist for suspected skin cancer found that the intervention increased referral sensitivity (conversion rate) and accuracy. In a report of two consecutive cancer audits in Scotland, the detection rate for all cancers in one Health Board area increased from 46% to 58% following an intensive educational intervention, although the quality of the data was poor and likely to be subject to ascertainment bias (Baughan *et al*, 2009).

As referral rates increased between 2010 and 2013, so conversion rates reduced from 11.4% to 10.2%. This inverse relationship has been previously described (Meechan *et al*, 2012) and reflects a predictable regression towards the risk of cancer associated with National Institute for Health and Care Excellence criteria for urgent referral, which is generally around 5%.

### Implications for practice and future research

The differential changes in referral practice found in this evaluation were small and arguably not clinically significant when viewed in the context of much larger overall changes during the same period. Influences to which all primary care clinicians were exposed, such as the growing public discourse about delays in cancer diagnosis and outputs from the wider NAEDI, are likely to have had a greater effect. Referral practice is only one means by which to assess quality of care, and future evaluation should consider measures of consultation frequency, use of diagnostic tests, time to referral and to diagnosis, stage at diagnosis and patient experience.

We are unable to say whether the activities promoted through this quality improvement programme have become part of the culture and clinical practice for GPs. On the basis of the experience of other large-scale initiatives in primary care (Audit Scotland, 2013), it is likely that sustained leadership and support with resources will be needed, together with an evolving range of actions that actively involve practices.

## Figures and Tables

**Table 1:** Profile of practices in each intervention group by patient list size, age, sex and deprivation quintile (where quintile 1 is least deprived and 5 is most deprived)

		**Practice demographics**																	
		**List size**				**Proportion aged 65+ years**				**Proportion male**				**Deprivation quintile**					
**Practice group**	**Number of practices**	**Small (<3000)**	**Medium (3001–6000)**	**Large (>6000)**	***P*****-value**	**Lowest 25% of Practices (<11.8%)**	***P*****-value**	**Highest 25% of practices (>19.9%)**	***P*****-value**	**Lowest 10% of practices (<48.0%)**	***P*****-value**	**Highest 10% of practices (>52.9%)**	***P*****-value**	**Least deprived**	**2**	**3**	**4**	**Most deprived**	***P*****-value**
Any specified activity	2495	10.9%	27.9%	61.1%		16.3%		27.5%		9.2%		5.0%		21.8%	20.5%	18.4%	20.5%	18.8%	
No specified activity	3991	17.5%	32.2%	50.3%	<0.001	19.5%	0.001	28.6%	0.313	7.4%	0.013	9.1%	<0.001	18.7%	18.9%	19.6%	19.1%	23.7%	<0.001
Practice plans, clinical audit and significant event analysis	287	9.4%	26.5%	64.1%	<0.001	12.9%	<0.001	31.4%	0.001	7.3%	<0.001	4.9%	<0.001	21.7%	21.0%	13.6%	28.0%	15.7%	<0.001
Practice plans and clinical audit	84	10.7%	26.2%	63.1%		22.6%		16.7%		14.3%		4.8%		8.3%	10.7%	27.4%	21.4%	32.1%	
Practice plans and significant event analysis	164	15.2%	25.0%	59.8%		12.8%		31.1%		6.1%		9.1%		20.1%	17.1%	16.5%	22.0%	24.4%	
Clinical audit and significant event analysis	379	10.9%	28.5%	60.6%		10.4%		31.6%		5.9%		3.2%		20.8%	21.6%	17.2%	23.2%	17.2%	
Practice plans	194	15.6%	31.8%	52.6%		16.7%		24.0%		8.3%		7.3%		17.5%	11.3%	14.9%	25.8%	30.4%	
Clinical audit	452	8.8%	28.1%	63.1%		21.0%		21.2%		12.6%		5.5%		23.7%	21.0%	20.1%	15.7%	19.5%	
Significant event analysis	230	11.8%	29.4%	58.8%		17.5%		33.3%		12.7%		3.1%		14.8%	27.4%	21.3%	20.0%	16.5%	
No practice plans, clinical audits or significant event analysis	4696	16.5%	31.5%	52.1%		19.2%		28.4%		7.6%		8.4%		19.9%	19.3%	19.5%	18.9%	22.4%	
Risk assessment tools	1548	11.1%	27.6%	61.3%	<0.001	16.6%	0.050	24.8%	0.001	8.4%	0.712	4.7%	<0.001	22.9%	20.0%	17.5%	22.2%	17.5%	<0.001
No risk assessment tools	4938	16.2%	31.5%	52.3%		18.8%		29.2%		8.0%		8.4%		18.9%	19.4%	19.6%	18.9%	23.2%	
Total	6486																		

**Table 2:** Reported use of any specified NAEDI activity; impact on referral metrics (all cancers)

				**Number of practices with a change in the same direction**	
	**Year to March 2010**^a^ **(CI) (pre)**	**Year to March 2013**^a^ **(CI) (post)**	**Change (CI)**	**Total**	**Significant**
**2WW referral rate**^b^ **(per 100 000)**					
All practices	1437.9 (1434.6, 1441.2)	1856.4 (1852.7, 1860.0)	418.5 (29.1%) (28.7, 29.5)	5497 (84.8%)	3473 (53.5%)
Any specified activity	1441.2 (1436.1, 1446.3)	1872.1 (1866.4, 1877.9)	**430.9 (29.9%) (29.3, 30.5)**	2143 (85.9%)	1431 (57.4%)
No specified activity	1435.5 (1431.2, 1439.8)	1845.1 (1840.4, 1850.0)	409.7 (28.5%) (28.0, 29.0)	3354 (84.0%)	2042 (51.2%)
**Conversion rate (%)**					
All practices	11.4 (11.4, 11.5)	10.2 (10.1, 10.2)	−1.3 (−1.4, −1.2)	3867 (59.6%)	550 (8.5%)
Any specified activity	11.5 (11.4, 11.6)	10.1 (10.0, 10.2)	−1.4 (−1.5, −1.2)	1512 (60.6%)	235 (9.4%)
No specified activity	11.4 (11.3, 11.5)	10.2 (10.1, 10.3)	−1.2 (−1.3, −1.1)	2355 (59.0%)	315 (7.9%)
**Detection rate (%)**					
All practices	43.9 (43.6, 44.1)	47.8 (47.5, 48.0)	3.9 (3.6, 4.2)	3882 (59.9%)	412 (6.4%)
Any specified activity	43.7 (43.4, 44.1)	47.9 (47.6, 48.3)	4.2 (3.8, 4.7)	1556 (62.4%)	167 (6.7%)
No specified activity	43.9 (43.7, 44.2)	47.6 (47.3, 47.9)	3.7 (3.3, 4.1)	2326 (58.3%)	245 (6.1%)
**Emergency presentation rate (%)**					
All practices	23.4 (23.2, 23.6)	21.1 (20.9, 21.2)	−2.3 (−2.6, −2.1)	3655 (56.4%)	255 (3.9%)
Any specified activity	23.2 (22.9, 23.5)	21.0 (20.7, 21.2)	−2.2 (−2.6, −1.8)	1417 (56.8%)	98 (3.9%)
No specified activity	23.5 (23.3, 23.7)	21.1 (20.9, 21.4)	−2.4 (−2.7, −2.0)	2238 (56.1%)	157 (3.9%)

Abbreviations: CI=confidence interval; GP=general practitioner; NAEDI=National Awareness and Early Diagnosis Initiative; 2WW=2-week wait.

a Years to December 2009 and December 2012 for emergency presentation rate

b For practice groups, referral rates are directly age-standardised using the 1976 European standard population weights, shown with 95% CIs based on the Gamma distribution (Fay and Feuer, 1997). For individual GP practices, results here are based on the crude referral rate.

**Table 3:** Change in variability of practice level rates reported by the interquartile range and a test for change in variance, by intervention group

**Practice group**	**Interquartile range**			
	**Year to March 2010**	**Year to March 2013**	**Change**	***P*****-value for change in variance**
**Standardised referral ratio**				
Any specified activity	49.6	39.4	−10.1	<0.001
No specified activity	52.9	46.6	−6.4	<0.001
Practice plans, clinical audit and significant event analysis	47.4	35.0	−12.4	0.022
Practice plans and clinical audit	63.7	43.7	−20.0	0.003
Practice plans and significant event analysis	49.9	45.9	−3.9	0.243
Clinical audit and significant event analysis	47.9	36.9	−11.1	0.020
Practice plans	49.5	44.9	−4.7	0.274
Clinical audit	47.6	39.3	−8.3	<0.001
Significant event analysis	52.4	37.9	−14.5	<0.001
No practice plans, clinical audits or significant event analysis	52.2	45.3	−6.9	<0.001
Risk assessment tools	49.2	42.0	−7.2	<0.001
No risk assessment tools	52.3	44.7	−7.7	<0.001
**Conversion rate**				
Any specified activity	6.7	5.6	−1.2	<0.001
No specified activity	7.0	5.8	−1.2	<0.001
Practice plans, clinical audit and significant event analysis	6.5	5.8	−0.8	0.079
Practice plans and clinical audit	7.4	5.7	−1.7	0.165
Practice plans and significant event analysis	6.3	4.5	−1.9	0.026
Clinical audit and significant event analysis	6.8	5.8	−1.0	0.028
Practice plans	7.4	6.3	−1.1	0.103
Clinical audit	6.5	5.0	−1.5	0.004
Significant event analysis	7.1	4.5	−2.6	<0.001
No practice plans, clinical audits or significant event analysis	6.9	5.7	−1.2	<0.001
Risk assessment tools	6.9	5.7	−1.2	<0.001
No risk assessment tools	6.9	5.6	−1.2	<0.001
**Detection rate**				
Any specified activity	18.2	15.6	−2.6	0.002
No specified activity	18.5	16.6	−1.9	<0.001
Practice plans, clinical audit and significant event analysis	17.8	16.4	−1.3	0.656
Practice plans and clinical audit	15.9	16.3	0.4	0.262
Practice plans and significant event analysis	13.6	14.4	0.9	0.726
Clinical audit and significant event analysis	18.9	14.9	−4.1	0.097
Practice plans	16.7	18.1	1.4	0.985
Clinical audit	16.7	14.4	−2.2	0.894
Significant event analysis	18.4	15.8	−2.6	0.006
No practice plans, clinical audits or significant event analysis	18.5	16.4	−2.1	<0.001
Risk assessment tools	18.1	15.6	−2.5	0.004
No risk assessment tools	18.5	16.5	−2.0	<0.001

	**Interquartile range**			
	**Year to December 2009**	**Year to December 2012**	**Change**	***P*****-value for change in variance**
**Emergency presentation rate**				
Any specified activity	12.2	11.3	−0.9	0.032
No specified activity	13.1	12.4	−0.7	0.013
Practice plans, clinical audit and significant event analysis	11.7	10.5	−1.2	0.154
Practice plans and clinical audit	10.8	10.9	0.1	0.776
Practice plans and significant event analysis	10.2	11.2	1.0	0.657
Clinical audit and significant event analysis	11.9	10.6	−1.3	0.961
Practice plans	14.7	10.9	−3.8	0.128
Clinical audit	12.8	12.7	−0.1	0.770
Significant event analysis	10.9	9.8	−1.1	0.106
No practice plans, clinical audits or significant event analysis	13.0	12.2	−0.8	0.003
Risk assessment tools	12.0	11.4	−0.6	0.086
No risk assessment tools	13.3	12.1	−1.2	0.006

**Table 4:** Factors found to be associated with success in quality improvement initiatives

Kaplan *et al* (2010)	This evaluation
	(+)=Facilitator
	(−)=Barrier
Leadership from senior management	(+) Clear and consistent communication, strong leadership from NCAT, efforts to engage all sites. Regular updates on progress
Supportive organisational culture, that is, open to change; values in line with those of proposed initiative)	(+) Shared values at network level (−) Perceived professional boundaries (−) Changes in organisational structure and function in a range of partner organisations, for example, PCTs to CCGs and Public Health moving into local authorities (−) Changes in professional roles (−) Public service bureaucracy
Data infrastructure and information systems	(+) Effective data linkage or alternative ways to exchange information (−) Systems that conflict between partner organisations or when unable to share data, for example, between CRUK and GPs
Previous involvement in quality improvement	(+) Previous existing relationships between participating organisations (+) New partnerships for some initiatives – generates enthusiasm
Physician involvement	(+) Clinical leadership (+) GP engagement (areas with good engagement were much more successful)
Microsystem motivation to change	(+) Some individual staff feel personal benefit as a result of changing (altruism or professional gain) (−) Some staff not motivated to change due to job/role not being secure (+) Shared understanding of NAEDI aims (+) Common belief in the value of NAEDI
Resources	(+) Some short-term capacity provision (−) Staff cuts (−) Some budget reductions (−) Short timescales
Team leadership	(+) Champions at team level (−) Some caution from leaders where teams were disrupted due to organisational change
Additional barriers affecting the success of NAEDI	(−) Multiple target populations (−) Wide scope of intervention (−) Concurrent internal and external change (−) NHS reform to structure and policy (−) NHS and Local Authority financial and employment legislation

Abbreviations: CRUK=Cancer Research UK; GP=general practitioner; NAEDI=National Awareness and Early Diagnosis Initiative; NCAT=The National Cancer Action Team; NHS=National Health Service.

## Box 1. NAEDI supporting primary care practice activities

**Clinical audit**

An audit of new cancer diagnoses (excluding screen-detected and non-melanotic skin cancers), usually for the previous 12 months. Practices use a standard audit template developed by and freely available from the Royal College of General Practitioners (RCGP).

**Significant event analysis**An approach to quality improvement that is well established in general practices. It involves a structured review by the practice team of all that happened in relation to an event of interest, which may be adverse, exemplary or simply important (in this case the diagnosis of cancer). It addresses the questions: What happened? Why did it happen? What can be learnt? What should be changed?

Practice cancer plans

A practice team activity, typically undertaken following review of the practice's cancer profile (a report of performance against 25 metrics for cancer screening and diagnosis provided annually by the National Cancer Intelligence Network). Guidance and a template for practice plans are freely available from the RCGP. The plan records key data related to current activity and helps to establish targets for the future. It then details the key tasks to be undertaken in order to improve cancer-related activities.

Risk assessment tools

These provide GPs with estimates of the risk of a specified cancer for individual symptoms, combinations of symptoms and for repeat consultation with the same symptom. Initially available as mouse mats and desk easels, they were widely distributed by NCAT. They were later provided to a limited number of practices on an electronic platform that interfaced with the practice clinical system.
