# Supplementary material for: Assessing the impact of an English national initiative for early cancer diagnosis in primary care
Source: Br J Cancer. 2015 Mar 3;112(Suppl 1):S57–64. doi: 10.1038/bjc.2015.43 (PMC4385977; doi:10.1038/bjc.2015.43)
Supplement: Supplementary Tables 1 and 2 [file bjc201543x1.doc]

**Appendix**

Table 1:Reported use of Risk Assessment tools; impact on referral metrics for lung cancer

Table 2: Reported use of Risk Assessment Tools; impact on referral metrics for colorectal cancer
