# Supplementary figures and images for: Assessing the impact of an English national initiative for early cancer diagnosis in primary care
Source: Br J Cancer. 2015 Mar 3;112(Suppl 1):S57–64. doi: 10.1038/bjc.2015.43 (PMC4385977; doi:10.1038/bjc.2015.43)

Figure 1 Logic model: Cancer Networks Supporting Primary Care


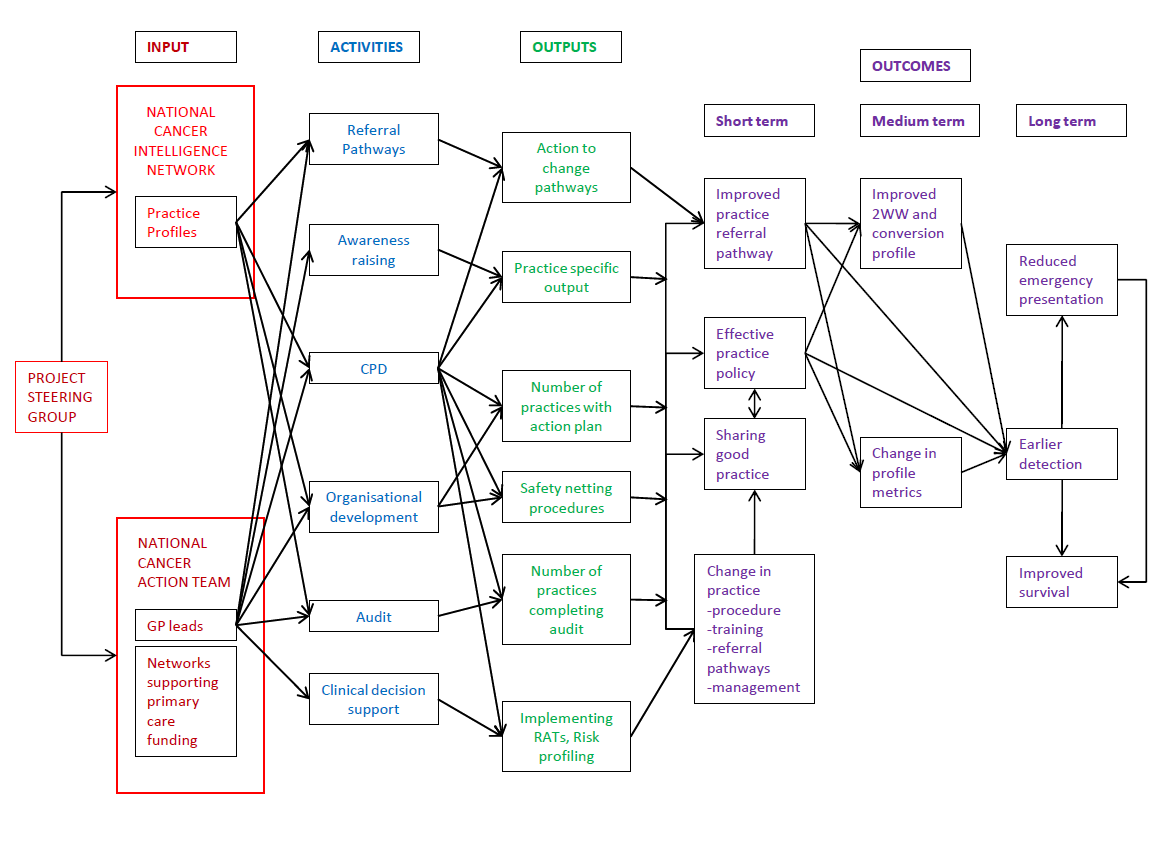

Supplement: Supplementary Figure 1 [file bjc201543x2.doc]
