# Supplementary material for: Assessing the impact of an English national initiative for early cancer diagnosis in primary care
Source: Br J Cancer. 2015 Mar 3;112(Suppl 1):S57–64. doi: 10.1038/bjc.2015.43 (PMC4385977; doi:10.1038/bjc.2015.43)
Supplement: Supplementary online material [file bjc201543x3.doc]

**Captions for Supplementary on-line material: National initiative for early cancer diagnosis**

Figure 1: Logic model: Cancer Networks Supporting Primary Care

Table 1:Reported use of Risk Assessment tools; impact on referral metrics for lung cancer

^ Years to December 2009 and December 2012 for Emergency Presentation rate

# For practice groups, referral rates are directly age-standardised using the 1976 European Standard Population

weights, shown with 95% confidence intervals based on the Gamma distribution (Fay and Feuer, 1997). For individual practices, results here are based on the crude referral rate

Table 2: Reported use of Risk Assessment Tools; impact on referral metrics for colorectal cancer
